# Hydroxyurea therapy for children with sickle cell disease: describing how caregivers make this decision

**DOI:** 10.1186/s13104-015-1344-0

**Published:** 2015-08-25

**Authors:** Susan Creary, Susan Zickmund, Diana Ross, Lakshmanan Krishnamurti, Debra L. Bogen

**Affiliations:** Division of Hematology-Oncology, Nationwide Children’s Hospital, The Ohio State University, 700 Children’s Drive Columbus, Columbus, OH 43205 USA; Department of Internal Medicine, University of Pittsburgh, Pittsburgh, PA USA; Division of Hematology/Oncology/BMT, Emory University School of Medicine, Atlanta, GA 30322 USA; Division of General Academic Pediatrics, Children’s Hospital of Pittsburgh, University of Pittsburgh School of Medicine, Pittsburgh, USA

**Keywords:** Hydroxyurea, Sickle cell, Decision making, Qualitative research

## Abstract

**Background:**

Hydroxyurea (HU) is underutilized in children with sickle cell disease (SCD) because caregivers frequently decline HU when it is offered. This study explores what impacts this decision.

**Results:**

Caregivers of children with clinically severe SCD whose children were offered HU previously were interviewed. We used a qualitative analytical approach to analyze their telephone interview transcripts. Caregivers who chose HU (n = 9) reported their children had severe SCD, sought detailed information about HU, and accepted HU as a preventative therapy. In contrast, caregivers who did not choose HU (n = 10) did not perceive their children as having severe SCD and did not question their child’s provider about HU.

**Conclusions:**

This study identifies specific areas that providers should address to when they discuss HU with families so that they can make informed decisions. Our study also uncovered factors that are important to consider when designing future interventions to improve hydroxyurea acceptance and when developing decision-aid tools to assist caregivers of children with SCD who are considering disease modifying therapies.

## Background

Sickle cell disease (SCD) is an inherited red blood cell disorder that leads to early morbidity and mortality [1, 2]. Hydroxyurea (HU) is the only available disease-modifying medication for patients with SCD [3]. Clinical trials show that HU decreases vaso-occlusive pain (VOC), acute chest syndromes (ACS), hospitalizations, and transfusions in both pediatric and adult patients [4, 5], and current guidelines are to offer HU therapy to all pediatric patients with Hemoglobin SS and Sβ^0^ SCD to reduce and prevent SCD complications [6].

HU is underutilized in clinical practice, and this results in worse clinical outcomes in pediatric patients with SCD. A major barrier to prescribing HU therapy is that 20 % of patients do not accept HU as a therapeutic option when it is offered [7]. Surveyed adult hematologists believe that this is because patients are fearful of side effects [8], whereas adult patients suggest that this is because patients lack knowledge about HU therapy and have unrealistic expectations about the time to effect with HU therapy [9]. For pediatric patients with SCD, caregivers are the primary decision makers but little is known about the factors that influence their decision about HU. Findings from caregiver surveys suggest that caregivers are concerned about HU’s side effects but also may not identify their child as sufficiently symptomatic to warrant HU therapy [10]. However, in-depth studies of caregivers that may identify significant, but unrecognized caregiver barriers do not exist. Fully understanding the caregiver perspective may help clinicians provide better education, counseling, and expectations to families about HU so that caregivers can make informed decisions for their children. It may also impact the design of future interventions to improve HU acceptance and decision-aid tools to assist caregivers who are considering other disease modifying therapies for SCD, such as chronic transfusion therapy and stem cell transplant. In this study we aim to explore what impacts caregivers’ decision to choose HU or not choose HU therapy for their children with SCD.

## Methods

### Participant identification

This study was approved by the University of Pittsburgh Institutional Review Board. The pediatric SCD team at Children’s Hospital of Pittsburgh (CHP) identified pediatric patients who were offered HU therapy for clinically severe SCD prior to March 2012. Caregivers were eligible to participate if they were ≥18 years of age and their child’s medical record confirmed that a SCD provider had offered HU at least once prior to enrollment. We mailed prospective caregivers recruitment letters that stated the purpose of the study. All caregivers who contacted the research team after receiving the letter were interviewed. We then attempted to contact the remaining prospective caregivers sequentially until thematic saturation was achieved. Caregivers received a $20 gift card for participating in this study.

### Qualitative interviews

**Fig. 1:**
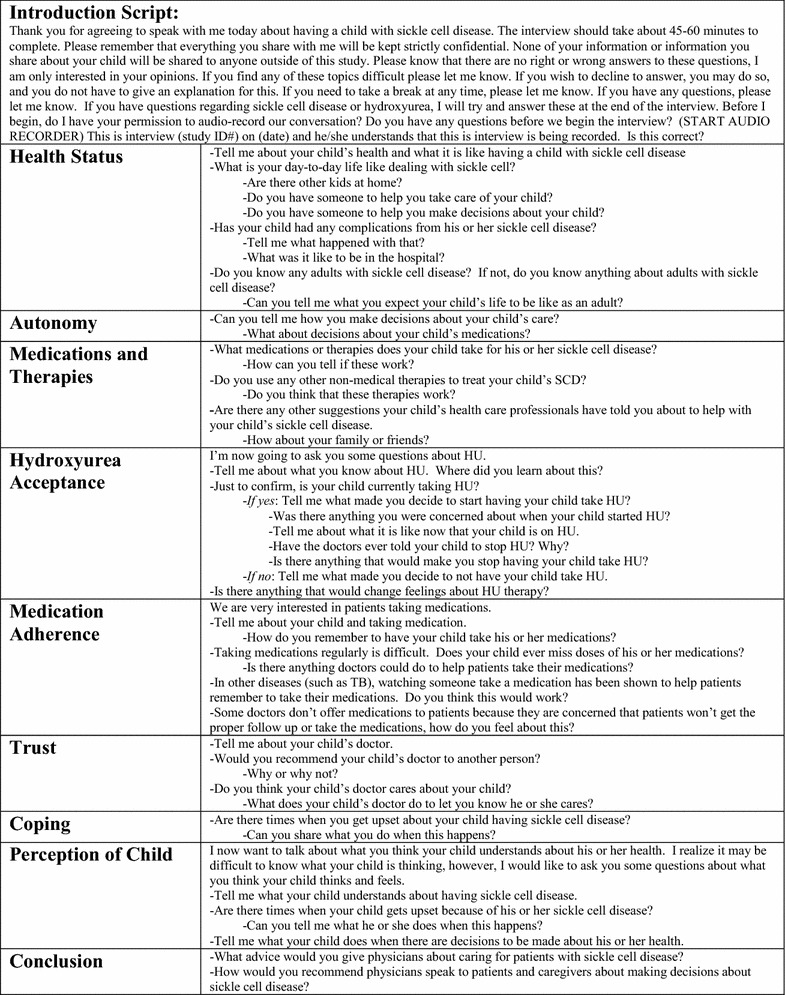
The semi-structured interview guide

The qualitative interviews followed a semi-structured interview guide. (Fig. 1) The interview guide was multidimensional, allowing the interviewer to explore different topical pathways, depending on how respondents answered prior questions. The specific categories that were chosen were based on a literature review of both HU and medical decision making [7–9, 11]. The initial interview guide was pilot tested on volunteers without SCD and the study’s qualitative expert (SZ) and then adjusted to improve the clarity and flow of the questions. The interviewer (SC) conducted all of the interviews over the telephone from March to October 2012. SZ trained SC to listen carefully to each interviewee’s responses to guide the interview spontaneously, avoid repetition, and probe interesting comments to investigate how caregivers made their decision to choose or not choose HU therapy when their child’s provider offered it. At the end of the interview, caregivers completed a short, verbal, demographic survey. A medical transcriptionist transcribed the digitally recorded interviews verbatim for analysis. SC reviewed these transcripts prior to the analysis for transcription accuracy.

### Codebook construction

Two trained coders (SC and DR) read the transcripts iteratively to develop a qualitative codebook of themes, using the qualitative editing style developed by Miller and Crabtree [12]. All transcripts were double coded and the coders met regularly to resolve any coding discrepancies.

### Medical record review

We used a standardized data collection form to record patients’ demographic information and the provider-documented indication for HU therapy within the medical record. We used descriptive statistics to describe demographic data.

## Results

We identified 52 patients with SCD at CHP who were offered HU for clinically severe SCD before March 2012. Nineteen caregivers of 16 patients completed the interview. Two caregivers contacted the research team after receiving the recruitment letter and then we consecutively contacted the remaining caregivers (n = 17) by telephone until thematic saturation was achieved. Each caregiver we contacted agreed to participate in the study. Approximately half (n = 9) of the interviewed caregivers chose HU for their children with SCD (Table 1). The interviews lasted 18–54 min in duration (median 29 min, and mean 32 min).

**Table 1:** Characteristics of children with SCD and their interviewed caregivers

Variables	Did not choose HU	Chose HU
Patients	n = 8	n = 8
Patient age (years)		
Median	13	8
Range	5–18	2–17
Gender (n)		
Female	4	3
Genotype (n)		
SS	5	8
SC	3	0
Indication for HU therapy (n)		
ACS only	1	2
Frequent VOC only	3	1
VOC and ACS	4	5
Caregivers	n = 10	n = 9
Caregiver age (years)		
Median	35.5	32
Range	26–67	24–45
Caregiver gender (n)		
Female	8	7
Caregiver relationship to patient (n)		
Biological parent	6	7
Biological grandparent	1	2
Other (adoptive parent or family friend)	3	0
Caregiver education (n)		
<12th grade	0	0
High school	1	1
Associates degree or finishing degree	4	5
Bachelors	4	2
Graduate degree	0	1
Did not disclose	3	0
Caregiver employed (n)		
Yes	5	8

During the iterative review of the transcripts, we identified common themes. Caregivers shared similar comments and views about how SCD’s impacts their daily life and fears that they had about initiating HU therapy. There were other themes where the two caregiver groups had more contrasting comments and views (i.e. caregivers’ SCD knowledge, caregivers’ perception of their child’s disease severity, caregivers’ interactions with their medical team, and experiences with HU).

### SCD’s impacts daily life

Most caregivers expressed difficulty living and coping with having a child with SCD. Many commented that SCD can be unpredictable and sometimes unrelenting. “Well there is never any rhyme or reason with sickle cell. We learned that over the years.” Others shared that having a child with SCD significantly affected the entire family because it frequently interrupted their familys’ plans, careers, and even their ability to make a living. One stated, “Oh, (the pain episodes) were overwhelming with dealing with his pain, and I have two other kids. So, it was really overwhelming, plus work. Like trying to get the strength down and you don’t know where you are going to get the strength from. But it works. I’ve prayed about it and prayed about it and I’ve prayed about it and that helps me get through it more than anything.” Caregivers admitted that their children had become emotionally upset because of their SCD, especially when the disease limited their activities or resulted in an emergency room visit or hospitalization.

### Fears about initiating HU therapy

Commonly, caregivers stated they were fearful about starting HU therapy, even if they ultimately decided to choose HU. They expressed anxiety and difficulty understanding why a child with SCD would be offered HU, especially when they learned it was a chemotherapy medication. “She (the nurse practitioner) was saying that they use it (HU) on chemo patients. My thing was like, ‘Well it’s not going to cause him to get cancer or anything like that?’ And she was like, ‘No, it is not that type of medication,’ and I was like, ‘Oh, okay.’ Because when you hear… when she was explaining to me the medication, when she was saying that this is what the chemo patients use, and I was like well, my first instinct was like, do we have cancer and you’re not telling me? That was my first instinct.”

### Caregivers’ SCD knowledge

When caregivers were asked what they felt was important for a physician to know when caring for patients with SCD, all caregivers commented that providers should give as much information to parents as possible to better prepare them to deal with their children’s health. Both groups indicated that they felt they would benefit from having more information about SCD and some suggested an education program for parents and patients could be beneficial.

Both caregiver groups had caregivers who achieved a variety of different education levels (Table 1). Responses from caregivers who chose HU suggested that they had a better understanding of SCD and that they specifically sought more detailed and in-depth information about SCD and HU. “I’m a parent. They (the providers) just gave me a pamphlet. The pamphlet doesn’t tell you everything… there’s more research behind the pamphlet… I think that’s very important to a parent to give their child medication.” In contrast, many caregivers who did not choose HU admitted during the interview that they had a poor understanding of SCD and what to expect in the future. “I don’t even know what’s going to happen when she turns 18 or becomes an adult. Like I don’t know how this runs from now to then.”

### Caregiver’s perception of SCD severity

All of the caregivers in the study had a child who had experienced a severe SCD-related complication, such as ACS or VOC (Table 1). Caregivers who did not choose HU, however, indicated that they would only consider HU if their child had more severe complications in the future. “I see her to be severe, (to have severe SCD), but I don’t see her to be as severe as some of the cases that I’ve seen.” A few caregivers in this group also tended to focus on their child’s short-term health. “More than likely, we’re just concerned about keeping him healthy and stuff for now.” In comparison, caregivers who chose HU identified medications, such as HU, as a way to prevent complications and to extend their child’s life. “They say that your life expectancy (with SCD) is up, like, when you are 42 years old. I know people who live way past 42 years old. So, there are other ways around it. You have to take care of yourself. You have to take your medication (HU).”

### Caregivers’ interaction with the medical team

All caregivers in this study identified themselves as a decision maker for their child and stated that they were responsible for their child’s health and medications. When asked if they trusted their child’s hematologist, most spoke positively about their relationship with their child’s physician and consistently stated they would recommend their child’s SCD team to other caregivers.

Although caregivers sought guidance from their families and their child’s health providers, caregivers who chose HU felt the need to be proactive and ask their child’s providers questions. “They (the doctors) are easy to talk to, which is good, but also as a parent, you have to ask questions. You know, if you don’t understand anything. You know, a closed mouth doesn’t get fed. So if you don’t ask, they’re not going to answer it for you.” In contrast, caregivers who did not choose HU depended on their child’s physician to direct the medical decision-making process for their child. “I don’t second guess anything because I know it’s best for his care. If they (the doctors) say to do something, then we do it.” Furthermore, some of these caregivers reported that when their physician offered HU to them as a potential therapy, they did not engage their child’s physician in a discussion about HU, even when they were fearful about what they had read or heard from other sources. “I was curious (about HU) and I came home and I got on the computer and there were so many things about it that kind of frightened me… There was just a lot and I’m thinking, ‘Wow, what type of medication is this?’” But when asked in follow up questioning if this caregiver had asked her provider about what she had read, she paused and said, “No, I didn’t.”

### Experiences with HU

Two caregivers who did not chose HU revealed during their interviews that their children had previously tried HU but discontinued it. They both admitted that their children took HU for less than 6 months and missed doses. Ultimately, they stated that they decided to discontinue HU because their children continued to have VOC, experienced side effects, and did not seem to benefit from HU. “If her (the patient’s) stomach was hurting too much, we would stop (HU) for a couple of days and then we’d say, ‘Ok let’s try it again’ and that type of deal. But it really was bad. It (HU) really made her stomach hurt a lot. She really did not enjoy taking it whatsoever.” One of these caregivers went on to state that her child’s experience with HU was so terrible that she would not consider HU therapy in the future, even if their child’s SCD-related symptoms were to worsen.

In contrast, we asked caregivers who chose HU to share what they would tell another caregiver of a child with SCD about HU. They consistently stated that they would recommend HU because their own children had fewer SCD-related complications and more energy with HU. “I’d strongly recommend that they give it (HU) a chance and see what it can do because I’m sure it doesn’t work the same in everybody, but you never know until you try.” A few of these caregivers also expressed that they would be reluctant to stop HU therapy under any circumstances because their child was doing so well.

## Discussion

HU is an efficacious medication for pediatric patients with SCD, yet many caregivers do not choose HU for their child when it is offered [7]. The most recent version of the National Heart, Lung and Blood Institute Guidelines recommends that providers “offer HU” to all children with severe SCD to encourage shared decision making about HU [6]. The caregiver perspective, however, is largely unknown, and pediatric hematologists and nurses may find this information useful to better counsel caregivers about HU and to improve HU utilization.

It is important to note that all caregivers reported that SCD negatively affects their daily lives. These comments are striking and highlight the need for more widespread use of efficacious therapies, such as HU, to mitigate SCD complications. We also found that even caregivers who choose HU for their children reported being fearful about initiating HU. To overcome this fear, we suggest that providers initiate early and ongoing discussions about HU with families. This gives families time to gather information about HU and allows providers the opportunity to address parental fears before severe SCD complications begin to manifest and persist in patients’ daily lives.

Also, our interviews suggest that knowledge among caregivers of children with SCD is quite variable. It is clear, however, that caregivers want more information about SCD, its complications, and HU therapy so that they can make informed decisions. We suggest that the first step is to formally assess caregivers’ health literacy since this is associated with health outcomes [13, 14] and then to assess caregivers’ SCD knowledge, potentially using the validated Sickle Cell Knowledge Questionnaire [15]. Once health literacy and SCD knowledge gaps are identified, targeted interventions such as group education sessions, which have been shown to successfully improve knowledge for patients with chronic diseases, could be used to inform caregivers [16].

All of the caregivers in this study had children who had experienced a severe SCD complication, but similar to Oyeku et al. [10], many caregivers who did not choose HU did not perceive that their child had severe enough SCD to warrant HU therapy. Some possible explanations for this are that caregivers may not understand that frequent VOC and ACS are severe complications, have other children with SCD to use as a comparison, or want to acknowledge that their child has severe SCD.

It is important to note that a few caregivers on this study who did not choose HU had children with Hemoglobin SC disease. Although patients with Hemoglobin SC SCD typically have a milder clinical phenotype compared to patients with Hemoglobin SS SCD, patients with Hemolglobin SC can still have severe disease manifestations and for these patients, many providers will offer HU since small studies show it can be beneficial [17]. Thus, we suggest that providers routinely update families each time their child has a severe SCD-related complication so they can accurately assess their child’s risk and disease severity, since as many as half of parents will rate that their child has mild SCD [18].

In contrast to prior studies [9, 10], caregivers in our study did not consistently state that HU side effects were an important factor in their decision to choose or not choose HU. The caregivers of the patients that discontinued HU therapy did comment that side effects were a problem. Our results and other surveys suggest that caregivers may still have concerns and misconceptions about HU therapy and its potential to cause malignancy [11]. To overcome these potential barriers, we suggest that clinicians anticipate these questions and specifically address these concerns in their early discussions about HU with families. For instance, physicians could provide all families with the same information on HU, such as the indication for wanting to start this therapy, the length of time to expect before HU may begin to reduce symptoms, the importance of regular HU adherence, the theoretical malignancy risk, and the common side effects they may encounter so that caregivers have fewer unanswered questions and unrealistic expectations about HU.

Our study has a few limitations. First, inherent in the qualitative study design, the data is limited to the information that subjects chose to share during the interview. To facilitate participant sharing, we did reassure caregivers of their privacy and anonymity prior to their interview. We acknowledge though that the first author [SC] created the interview guide, completed the interviews, and was a hematology fellow who intermittently cared for some of the patients around the time that the interviews were completed and this may have limited the information that some caregivers chose to disclose. Second, three of the interviewed caregivers were from the same family. Although it is reasonable to expect that caregivers from the same family would report similar answers in the interview, each caregiver’s interview was conducted independently and privately so that caregivers could openly share their opinions even if they were different from other caregivers within the same family. To prevent over-representation of one family’s opinion in our results, we continued to interview caregivers of independent patients sequentially until thematic saturation was achieved. Lastly, although CHP is a tertiary children’s hospital with similar resources and a similar patient population to other pediatric SCD Comprehensive Programs in the United States, all of the caregivers we interviewed had children who were followed at a single institution and may limit the generalizability of our results.

## Conclusions

This study is unique as it provides an in-depth perspective about caregivers’ decision to have their children take or not take HU. Our results suggest areas where interventions could be developed (e.g. caregivers’ SCD knowledge or caregivers’ interactions with the medical team) and tested to determine if they result in increased HU use and improved outcomes in pediatric patients with SCD. These results could also be used when developing decision aid tools so that all the factors identified by caregivers as potential barriers are addressed and caregivers feel empowered and informed to make a decision about HU and other disease modifying therapies [19]. Finally, although most pediatric hematologists and published guidelines support that HU therapy be offered to all pediatric patients with Hemoglobin-SS and Sβ^0^ SCD, a consensus that HU is a standard-of-care treatment for children with SCD may provide the necessary reassurance to caregivers that HU is effective and safe and may result in more caregivers choosing HU for their children.

## Abbreviations

HU: hydroxyurea
SCD: sickle cell disease
VOC: vaso-occlusive pain
ACS: acute chest syndrome
CHP: Children’s Hospital of Pittsburgh
